# Screening Olive Leaves from Unexploited Traditional Greek Cultivars for Their Phenolic Antioxidant Dynamic

**DOI:** 10.3390/foods7120197

**Published:** 2018-12-03

**Authors:** Vassiliki T. Papoti, Maria Papageorgiou, Konstantina Dervisi, Evangelos Alexopoulos, Konstantinos Apostolidis, Dimitrios Petridis

**Affiliations:** 1 Department of Food Technology, Alexander Technological Educational Institute of Thessaloniki, GR-57400 Thessaloniki, Greece; tatina1989@windowslive.com (K.D.); v-alex@windowslive.com (E.A.); apastolandas@gmail.com (K.A.); petridis@food.teithe.gr (D.P.); 2 Department of Food Science & Technology, Perrotis College, American Farm School, GR-57001 Thessaloniki, Greece

**Keywords:** olive leaves, cultivar, phenols, flavonoids, antioxidants, *Olea europaea* L.

## Abstract

Quality characteristics of olive products significantly depend on cultivar (cv), among other factors. In this study, seven traditional, noncommercial Greek cultivars, along with the commercial Spanish Arbequina cv., were examined for the phenolic antioxidant dynamic of their leaves. Polar extracts (aqueous, methanol, and ethanol) were analyzed for Total Phenol (TP), Flavonoid (TFL), Hydroxycinnamic Acid Derivatives (THAD), Flavonol (TFLVN) contents, DPPH radical scavenging ability, and Ferric Reducing Capacity (FRAP). Selective characteristics of olive leaf methanol extracts for all cultivars were re-examined on a second sampling period. Olive leaf is considered a rich source of phenolic antioxidants total phenol content reaching 29.3 ± 1.3, 30.6 ± 0.4, and 27.0 ± 1.1 mg caffeic acid/g dry leaf for aqueous, methanol, and ethanol extracts, respectively) and all cultivars were considered of equal bioactive dynamic. TP data derived from Folin–Ciocalteu and another spectrophotometric assay employed presented a high correlation for all examined cases (*R*^2^ = 71.5–86.9%). High correlation (*R*^2^ = 0.92) was also found between TP and FRAP findings of aqueous extracts. Olive leaf is considered a promising source of phenolic antioxidants irrelevant to cultivar and therefore even cultivars less effective for oil or table olive production could be efficiently exploited for the bioactive dynamic of their leaves.

## 1. Introduction

The culture and significance of the olive tree in Mediterranean, and particularly in Greece, is manifested in many ways (documentary and Linear B tablets evidence, iconographic representations, archaeological evidence, paleobotanical remains, molecular analysis evidence, mythology, ethnobotanical information, literature, and customs) and dates back to prehistoric times. The olive tree is one of the earliest horticultural fruit trees whose products were and still are a part of religious rituals and ceremonial activities, symbols of peace, prosperity, and victory, used for aromatic oils and ointment preparation, as pharmaceuticals, cosmetics, food consumption, and other daily needs (lighting and heating) [1,2].

Today the higher living standards and demand for a healthier life style and extended life expectancy renewed the interest for olive products [3]. The well documented health related benefits of olive products, mainly due to their polyphenolic constituents, have launched the interest of consumers, industries (food, medicinal, and cosmetic) and scientists. EFSA’s claim [4] on the protection of blood lipids from oxidative stress and the related disorders from olive oil polyphenols intake (at least 5 mg of hydroxytyrosol and its derivatives per day) initiated the marketing of relevant products. 

Olive leaf is a byproduct of olive cultivation and processing resulting from tree pruning (~25% by weight), drupes collection for oil (up to 10% of olives total weight arriving at mills), and table olives production. It is considered an exceptionally cheap natural source of high-value-added compounds [3,5,6]. As a material, it has a long history of traditional use due its medicinal and bioactive properties [7,8], many of which have been verified from current scientific data [3,9,10]. Olive leaves contain high quantities of a large variety of phenolic bioactive compounds, similar to those present in olive oil, table olives, and other olive materials. Among the bioactive compounds of olive leaves are simple phenols (e.g., hydroxytyrosol), flavonoids (flavones, flavanones, flavonols, and 3-flavanols), and secoiridoids (e.g., oleuropein and derivatives), which show well-documented biologic activities including antioxidant, antimicrobial, anti-inflammatory, anticancer, and neuroprotective [3,5,6,10,11]. Today, there is still a huge potential for extended valorization of olive leaves for the production of pharmaceuticals, cosmetics, nutraceuticals and functional foods [3,6]. Available literature data deal mainly with commercial varieties, while varieties of low productivity in the oil and table olives sectors and of ornamental use, have not been studied. 

One of the most important factors that significantly affect phenolic content and composition is cultivar [3,12]. Today, more than 2000 cultivars are spread throughout olive growing regions. A large number of ancient cultivars still exist, but only a few are being cultivated for commercial reasons. The different olive cultivars present diverse morphological and physiological characteristics resulting in different uses and product qualities [13,14,15].

Greece is the third largest olive oil producer in the world, it is among the world’s top table olive producers while it possesses more olive varieties than any other country (~100) [14,15]. Its favorable olive tree environment results in products with exceptional quality characteristics. More than 70% of oil produced is extra-virgin [16] and Greek table olives hold an exceptional phenolic antioxidant potential [17,18]. 

In the present study, seven native but noncommercial Greek olive cultivars (since they are of low dynamic for oil and table olives production or they are used as ornamentals) are employed and the phenolic antioxidant potential of their leaves is comparatively examined. Olive leaf polar extracts (aqueous, methanol, and ethanol) are analyzed for their total phenol, flavonoid, hydroxycinnamic acid derivatives and flavonols contents, as well as 1,1-diphenyl-2-picrylhydrazyl (DPPH) radical scavenging ability and ferric reducing capacity. Selective characteristics of olive leaf extracts are re-examined during a second time period. The study aims to check the potency of profitable exploitation of olive leaves coming from widely distributed in Greece noncommercial cultivars to boost local income and benefit consumers, industries, markets, and society. 

## 2. Materials and Methods 

### 2.1. Materials 

Caffeic acid (98%, CAF) was purchased from Riedel de Haën (Seelze, Germany). Gallic acid (99.5%, GAL), DPPH radical (1,1-diphenyl-2-picrylhydrazyl, 90%), 2,4,6-tripyridyl-*s*-triazine (TPTZ), FeCl_3_·6H_2_O, and quercetin (QUERC) were purchased from Sigma Chemical Co. (St. Louis, MO, USA). Folin–Ciocalteu reagent, AlCl_3_, and Νa_2_CO_3_ (99.8%) were from Panreac Quimica (Barcelona, Spain). HPLC-grade methanol (MeOH) and ethanol (EtOH), as well as acetic acid were from Merck (Darmstadt, Germany). All other common reagents and solvents were of the appropriate purity from various suppliers. The water used was deionized, obtained by an ion-exchange resin system (ZALION 2000, IONEL, Athens, Greece) with a minimum resistance of 800,000 Ω/cm.

### 2.2. Plant Material 

Olive leaf samples were collected on two sampling dates (1 December 2012 and 10 December 2013) following fruit harvest in the last ten days of November. The examined cultivars were the representative and widely known Arbequina Spanish cv., and seven Greek traditional but unexploited ones, namely Asprolia, Atsilochou, Chrysophilli, Veroia, Gigas, Petrolia, and Pikrolia. Selective characteristics of the employed cultivars are summarized in Table 1 [14]. Trees were of approximately the same age (25–30 years old), cultivated under the same agricultural practices (no fertilization, average irrigation applied per drop, 1–2 times every 15 days from May to September with average water 2500–3000 m^3^/ha) in the same orchard (Troizina, Poros Island, Greece; coordinates: 37.505849° N, 23.406250° E, 5 m above sea level). The microclimate of the location is characterized by a mean average yearly rainfall of 480.7 mm; the months November to January account for 50% of total rain. The lowest mean average temperature is observed in February (8.4 °C) while July is the hottest month with a mean average temperature of 27.3 °C. The winds are north for most of the year with the exception of the months April to June and September when they turn south. Each sample corresponded to leaves collected by hand from same height branches of the whole perimeter of 5 trees for each cultivar. The leaves from the 5 trees were combined into a representative batch (~0.5 kg), which was used to contact analysis in triplicate. Samples collected had completed their developmental stage (mature leaves) and trees employed had reached their optimal maturity. Leaf material was immediately transferred in the lab, washed with deionized water (dH_2_O), and lyophilized (Christ, Gamma 1–20, Germany). Dried olive leaf samples (OLF) were placed in dark containers and stored in desiccators in a cool dark place at room temperature. Analysis was performed within a brief time period. 

### 2.3. OLF Extracts’ Preparation

OLF (2%, *w/v*) was treated in an ultrasonic bath (Elmas 30H Elmasonic, Singen, Germany; ultrasonic power effective 80 W, ultrasonic frequency 37 kHz) at room temperature for 15 min. Extraction solvents were water, ethanol, or methanol. OLF extracts were prepared in triplicate and each preparation was further analyzed. 

### 2.4. Content in Total Phenols Determined via the Folin–Ciocalteu Assay (TPFC) 

The assay employed was according to that described elsewhere [12]. Suitable aliquots (0.25 mL) of OLF extracts were transferred into a 10 mL volumetric flask. Subsequently, 5 mL of water and 0.5 mL of the Folin–Ciocalteu reagent were added. After 3 min, 1 mL of saturated (35%, *w/v*) sodium carbonate solution was added and the mixture was then diluted with water to 10 mL. One hour later the absorbance was measured at 725 nm against a blank solution. Calibration curves were constructed using standard solutions of CAF and TPFC content was expressed as mg CAF/g dry leaf.

### 2.5. Content in Total Flavonoids (TFL)

The TFL was determined according to Cvek et al. [19] with slight modifications. An aliquot (0.1 mL) of an aluminum chloride solution (2% aluminum chloride in 95/5 MeOH/acetic acid, *v/v*) was added to an aliquot of OLF extract (0.3–1 mL), and subsequently 1.4 mL of MeOH/acetic acid mixture (95/5, *v/v*) was added. The mixture was left for 30 min at room temperature, and thereafter the absorbance was measured at 415 nm against the control. Absorbance measurements were corrected by subtracting initial sample absorbance at 415 nm. Calibration curves were constructed using standard solutions of QUERC and TFL results were expressed as mg QUERC/g dry leaf. 

### 2.6. Antioxidant Activity Determined via the DPPH Assay 

The ability of methanolic and ethanolic OLF extracts to scavenge the DPPH radical was determined according to Nenadis and Tsimidou [20] with some modifications: 2.9 mL of a DPPH^•^ solution (0.1 mM in MeOH or EtOH) was mixed with 0.1 mL of methanolic or ethanolic OLF extract. The absorption at 516 nm (A516) was recorded at the beginning of the reaction (*t* = 0) and after 20 min (*t* = 20). The results were expressed as % Inhibition = ((A516 (*t* = 0) − A516 (*t* = 20)) × 100/A516 (*t* = 0)). 

### 2.7. Antioxidant Activity Determined via the FRAP Assay 

The FRAP assay was carried out according to Benzie and Strain [21] with some modifications. A mixture containing 3 mL of freshly prepared and prewarmed (at 37 °C) FRAP reagent and an aliquot of OLF aqueous extract was incubated at 37 °C for 20 min and the absorbance was then recorded at 593 nm. The ferric reducing ability of the examined extracts was assessed as CAF equivalents via the use of a respective calibration curve. 

### 2.8. Content in Total Phenols (TPHCl), Hydroxycinnamic Acid Derivatives (THAD), and Flavonols (TFLVN) 

Determination of bioactive phenolic classes was carried out according to Obied et al. [22] with slight modifications: 0.5 mL of OLF extract (ethanol, methanol, aqueous) was mixed with 1 mL 0.1% HCl–ethanol solution (0.1 mL HCl per 100 mL 95% ethanol) and 8.5 mL 2% HCl–ethanol solution. The absorbance of the mixture was measured after 20 min at 280, 320, and 360 nm to evaluate total phenols (TPHCl), hydroxycinnamic acid derivatives (THAD), and flavonols (TFLVN) expressed as mg GAL, mg CAF and mg QUERC/g dry leaf, respectively, through the corresponding calibration curves.

### 2.9. Statistical Analysis

Experiments were carried out in triplicate and results were expressed as mean ± standard deviation for every set of data and each sampling period examined. Data were analyzed using one-way analysis of variance (ANOVA), (*p* < 0.05); pair-wise Tukey’s Honestly Significant Difference test of means was also performed. Statistical analysis was carried out using JMP 13.1 (2016, SAS Institute Inc., Cary, NC, USA) and Minitab 18 (2017, Minitab Inc., State College, PA, USA). Variables and varieties were subjected to two-way hierarchical cluster analysis to detect meaningful groups. In the same set of data Principal Component Analysis (PCA) was also employed to detect particular relationships. 

## 3. Results and Discussion

### 3.1. Total Phenol Content of Olive Leaf Polar Extracts 

**Figure 1:**
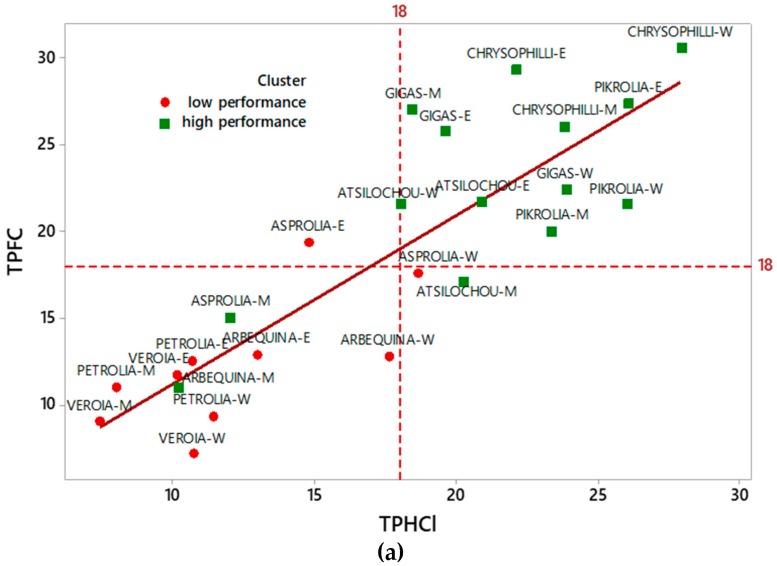

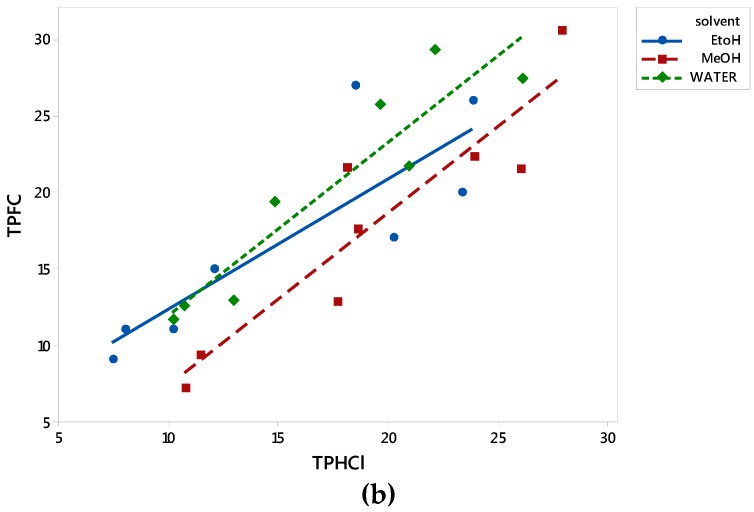
Correlation between TPFC and TPHCl data for all examined extracts. (**a**) W-indicates aqueous, M-methanol, and E-ethanol OLF extracts, respectively. (**b**) Data for aqueous, methanol and ethanol OLF extracts are depicted with green dashed line fitted in rectangles, red dashed line fitted in squares, and blue solid line fitted in circles, respectively. TPFC, total phenols determined via the Folin–Ciocalteu assay; TPHCl, total phenols determined via the alternative spectrophotometric assay; OLF, olive leaf samples.

Total phenol content was determined by two protocols; the widely employed Folin–Ciocalteu (TPFC), which is quite laborious, expensive, and time-consuming and an alternative (TPHCl) spectrophotometric one [22]. The latter, which generally enables also the simultaneous determination of other bioactive classes (total hydroxycinnamic acid derivatives, flavonols, and anthocyanins), is easier, less expensive, and quicker. The results of the two methods (*y* = TPFC and *x* = TPHCl) employed for total phenol content determination correlated very well for all analyzed extracts as is depicted in Figure 1. The equations of correlation for all the extracts together, and the individual ones (aqueous, methanolic, and ethanolic) were *y* = 1.532 + 0.970*x* (*R*^2^ = 0.741), *y* = 0.566 + 1.136*x* (*R*^2^ = 0.869), *y* = −3.964 + 1.131*x* (*R*^2^ = 0.853), and *y* = 3.824 + 0.8533*x* (*R*^2^ = 0.715), respectively. The high *R^2^* revealed that the alternative protocol can be efficiently employed for the phenol content determination of olive leaf polar extracts in a quick, easy and cheap way, especially in the case of a large number of samples, avoiding the labor of the Folin–Ciocalteu protocol. The efficient use of this protocol for TP content determination has been also employed in olive leaves and other natural products by others [23,24,25,26]. Goulas and Manganaris [26] also report a high correlation (*r* = 0.984) between the two methods for strawberry extracts. 

**Figure 2:**
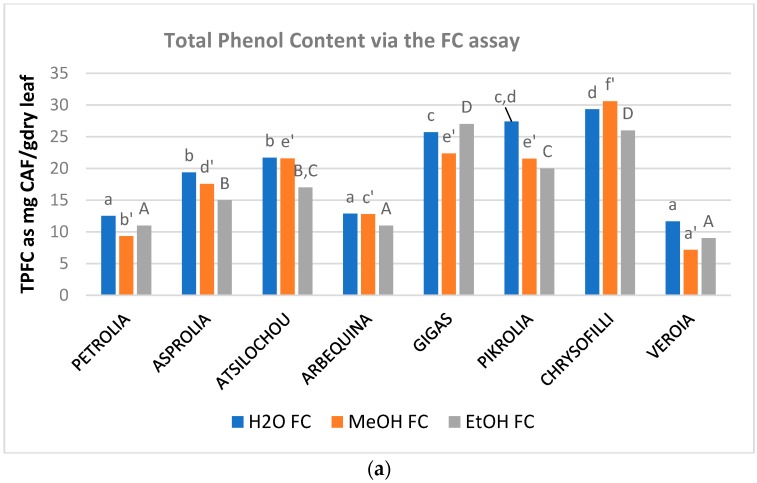

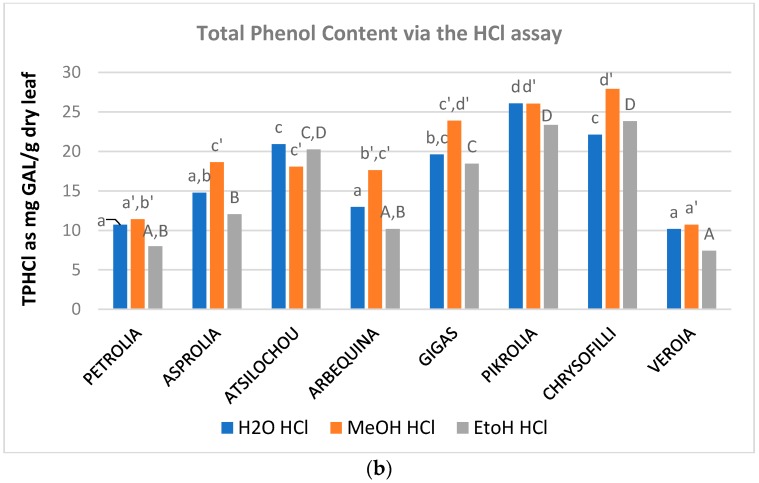
Total phenol content of OLF polar (aqueous, methanol, and ethanol) extracts as determined via the (**a**) Folin–Ciocalteu (TPFC) and (**b**) spectrophotometric by Obied et al. [22] assay (TPHCl); different letters (a–d, a’–f’, A–F) indicate statistical significant differences.

The phenol content (Figure 2, Table A1) of the examined aqueous, methanol, and ethanol OLF extracts ranged from 12 to 29, 7 to 31, and 9 to 27 mg CAF/g dry leaf as determined via the TPFC method and 10–26, 11–28, and 7–24 mg GAL/g dry leaf as determined via the TPHCl assay. The varieties analyzed showed almost similar trends in the three solvents employed and were therefore classified as being of higher (Chrysophilli, Pikrolia, Gigas, and Atsilochou) and lower (Asprolia, Arbequina, Petrolia, and Veroia) phenolic dynamic (Figure 1a and Figure 2). TP results were of the same size with those reported by other studies [25,27,28,29], although differentiations in sample treatments and experimental approaches do not always allow direct comparison of data. OLF polar extracts prepared under similar procedure from samples of 10 Greek cultivars (different than those examined in the present study, commercial ones, and from different sampling periods) possessed 5–19 mg CAF/g dry leaf [12,30]. Once more, olive leaf material was shown to be an exceptional source of phenols, with a content higher than that of other bioactive natural products (such as fruits, vegetables, nuts, spices, cereals, and grain legume seeds) [31,32].

### 3.2. Content of OLF Polar Extracts in Total Hydroxycinnamic Acid Derivatives (THAD), Total Flavonols (TFLVN), and Total Flavonoids (TFL)

**Figure 3:**
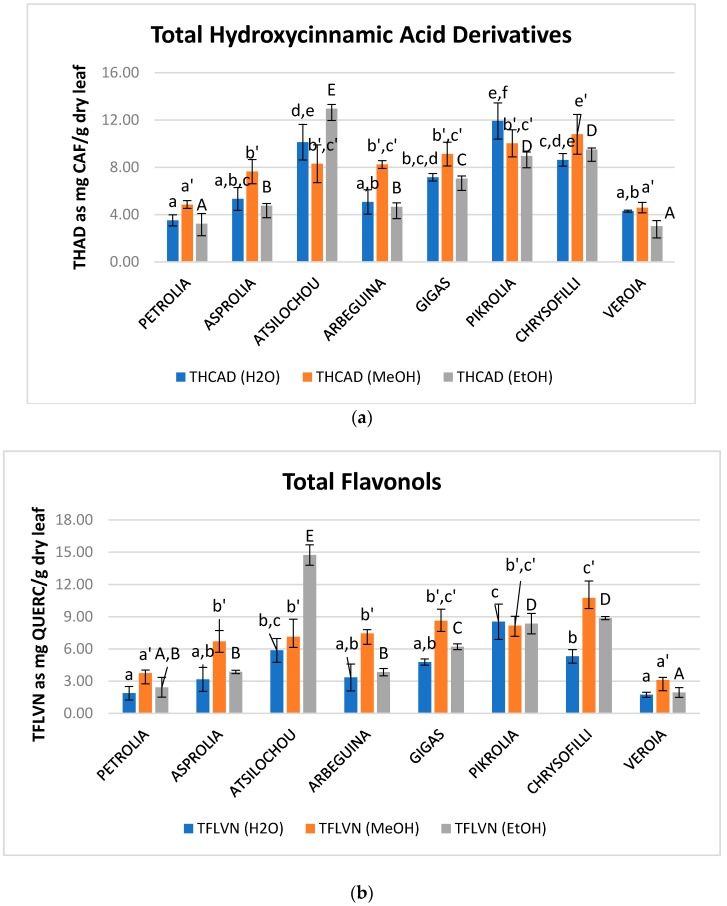

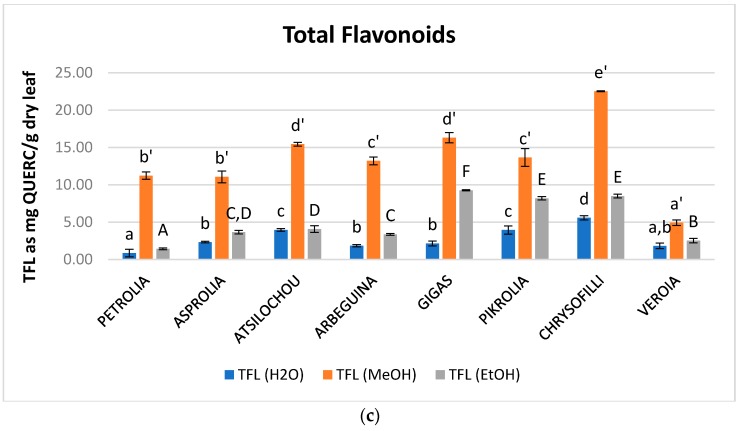
Content in total (**a**) hydroxycinnamic acid derivatives (THAD), (**b**) flavonols (TFLVN), and (**c**) flavonoids (TFL) of aqueous, methanol, and ethanol olive leaf (OLF) extracts; different letters (a–f, a’–e’, A–F) indicate statistical significant differences.

Flavonoids are considered an important fraction of olive leaf phenolics contributing to an important extend in the antioxidant activity of its extracts [23]. Total flavonoid content of the examined extracts ranged from 0.9 to 5.6, 4.9 to 22.5, and 1.4 to 9.3 mg QUERC/g dry leaf for water, methanol, and ethanol, respectively (Figure 3, Table A1). MeOH extracts presented significantly higher FL content for all examined cultivars in comparison to ethanol and aqueous ones (Figure 3c). Many of the flavonoids that are expected in OLF are found in their glycosidic form [11] and this form is known not to achieve high ethanol levels for extraction [33]. Moreover, water is considered less preferable for the extraction of flavonoids from OLF [5] while flavonols are also expected to present low solubility in this solvent [34]. On the other hand, methanol has been reported to preferable extract flavonoids from natural products when compared to water and ethanol [35,36,37,38]. Methanol extracts of the present study presented similar values with the methanol extracts studied in another study [24] (up to 26.5 mg QUERC/g dry material) although extraction conditions and the employed analytical protocol are different. 

Hydroxycinnamic acid derivatives are also considered an important fraction of olive leaf material since they comprise compounds such as verbascoside and caffeic, ferulic, coumaric, chlorogenic acids, that are considered to contribute noticeably to the activity of OLF extracts [23,39,40]. Regarding flavonols, quercetin and rutin [3,11,39] are mainly expected in olive leaves and are also considered to be related with health benefits [41,42] and contribute to the bioactive character of OLF [23,40]. THAD and TFLVN values of examined extracts: EtOH: 3–13, MeOH: 5–11, H_2_O: 4–12 mg CAF/g dry leaf and EtOH: 2–15, MeOH: 3–11, H_2_O: 2–9 mg QUERC/g dry leaf, respectively (Figure 3a,b, Table A1). Atsilochou ethanol extracts presented significantly high THAD and TFLVN values (Figure 3a,b, Table A1). Methanol extracts of the present study exhibit similar THAD and TFLVN values with those reported by Putnik et al. [24] (up to 2.6 mg CAF and 9.6 mg QUERC/g dry material, respectively). Moreover, the ethanol extracts of the studied low dynamic varieties (Petrolia, Veroia, Asprolia, and Arbequina) presented similar THAD and TFLVN values with those examined by Tsakona and collaborators [25] (2.1 mg CAF/g dry leaf and 3.8 mg QUERC/g dry leaf). The THAD and TFLVN values of the studied olive leaf extracts were of the same size to those reported for strawberry [26] and aromatic plant (*Salvia fruticosa* and *Origanum dictamnus* L.) extracts [25] examined under similar methodologies. 

Total flavonoid and total flavonol contents were better correlated in the case of methanol (*R*^2^ = 0.82) and less in the case of water (*R*^2^ = 0.53). Regarding ethanol extracts no correlation was shown (*R*^2^ = 0.19). Although total flavonoids should include total flavonols, the values of the latter were not always lower than that of the former, which was attributed to the different principles of the employed protocols. It is known that analytical parameters employed even for the same determination (e.g., total flavonoid content) can even alter the order of the examined samples in terms of the measured value [37]. 

### 3.3. Statistical Analysis Data of the TP, THAD, TFL, and TFLVN Parameters 

**Figure 4:**
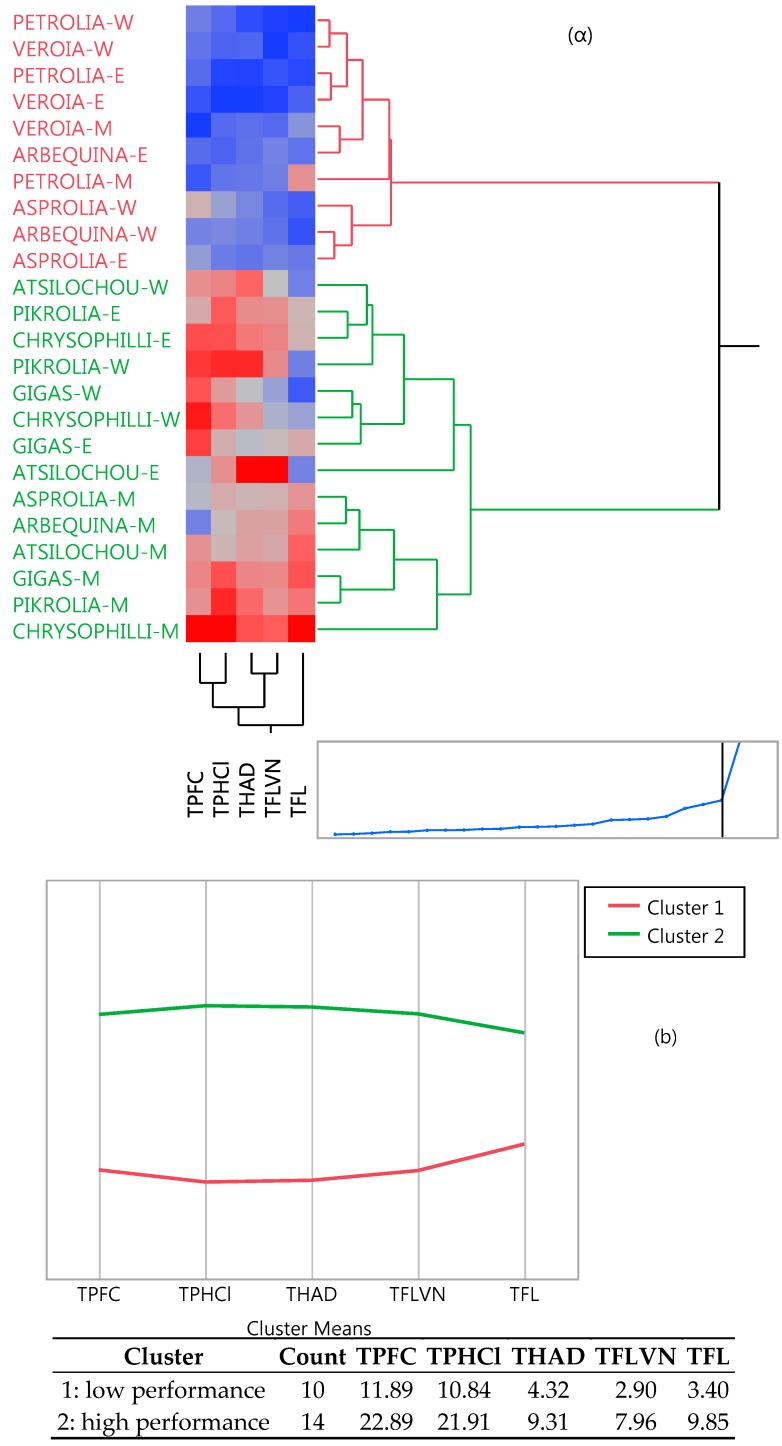
Two-way clustering dendrogram showing (**a**) the position of cultivars in the two clusters and (**b**) the intensity and means of the studied parameters; red and green denotes low and high performance clusters, respectively.

**Figure 5:**
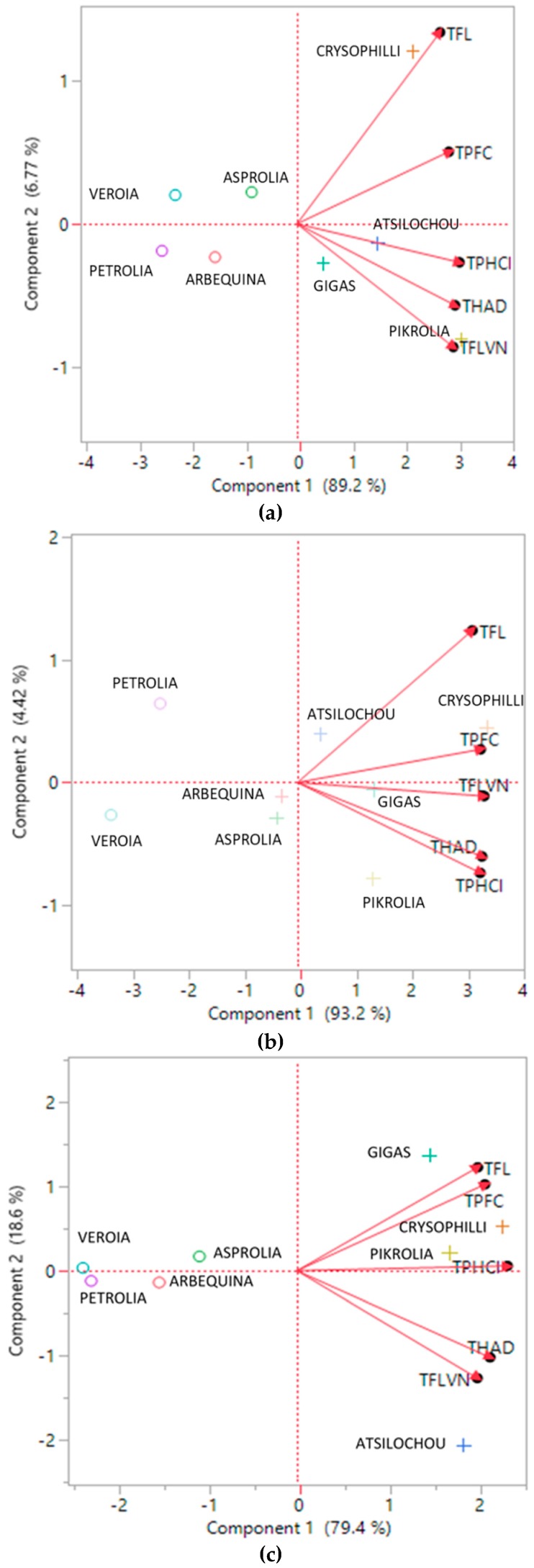
PCA biplots summarizing the relationship between the studied parameters and the olive leaf varieties. (**a**), (**b**), and (**c**) depict data from the aqueous, methanol, and ethanol extracts, respectively. TPFC, total phenols determined via the Folin–Ciocalteu assay; TPHCl, total phenols determined via the alternative spectrophotometric assay; THAD, total hydroxycinnamic acid derivatives; TFLVN, total flavonols; TFL, total flavonoids.

Cluster analysis (Figure 4) revealed two distinctive groups, one of lower and one of higher performance, as the dendrogram and the means of the table and graph clearly show. High cluster group means were two to three-fold higher than those of the low cluster group. The same clusters are also depicted in Figure 1a (in which two and one misfits are found respectively in the low and high performance cluster). Moreover, the same classification can be also shown from the PCA data (Figure 5), since the low performance varieties are illustrated on the left side and the high ones on the right side of the graphs. Furthermore, from the same PCA graphs the correlation between the studied parameters can be determined; parameters showing angles with low apertures indicate a strong positive relationship, while those with vertical apertures do not correlate at all. 

### 3.4. Antioxidant Activity of Olive Leaf Polar Extracts 

**Figure 6:**
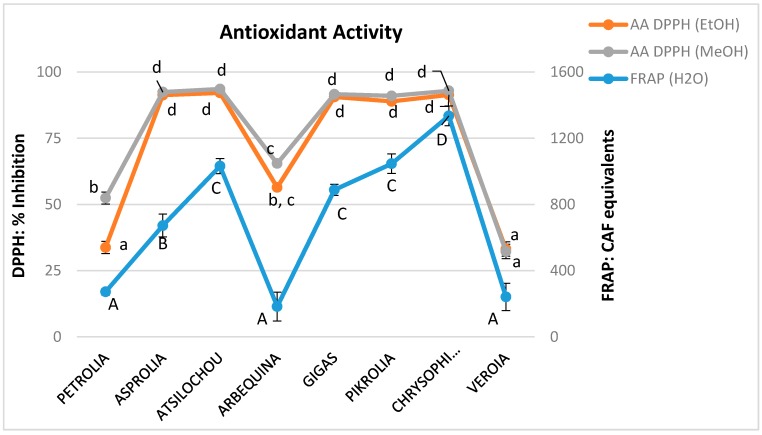
Antioxidant activity as determined via the 1,1-diphenyl-2-picrylhydrazyl (DPPH) assay, expressed as % DPPH radical scavenging inhibition for the methanol and ethanol extracts as well as ferric reducing capacity (FRAP), expressed as CAF equivalents (mg/L) for the respective aqueous extracts; different letters (a–d, A–D) indicate statistical significantly differences.

**Figure 7:**
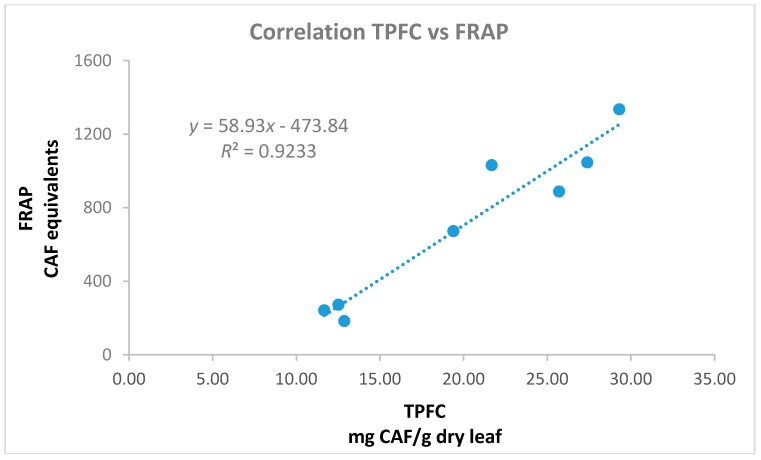
Correlation of total phenols determined via the Folin-Ciocalteu assay (TPFC, mg CAF/g dry leaf) and ferric reducing capacity (FRAP, mg CAF/L) data of the aqueous olive leaf extracts.

The antioxidant activity of the materials was examined by the application of the FRAP assay in the aqueous olive leaf extracts and the DPPH radical scavenging activity in the alcoholic extracts. The methods employed are commonly applied for the analysis of many matrices [43] such as nuts [44], vegetable oils [45], fruits, and relevant products [26,46,47,48]. All the examined polar extracts (aqueous, methanol, and ethanol) presented similar trends (Figure 6, Table A1). Those with higher TP content showed remarkable antioxidant performance, when lower activity was shown for extracts with inferior TP values as has been expected and discussed by other studies on olive leaves [49]. Methanol and ethanol extracts possessed almost identical DPPH radical scavenging activity (although their TP, THAD, TFLVN, and TFL values presented statistical significantly differences), dividing cultivars in stronger (Chrysophilli, Gigas, Pikrolia, Atsilochou, and Asprolia; MeOH: 91–94%, EtOH: 89–92% inhibition) and weaker (Arbequina, Petrolia, and Veroia; MeOH: 32–65%, EtOH: 33–56% inhibition) scavengers. Additionally, Chrysophilli, Gigas, and Pikrolia were distinguished for their ferric reducing ability, while Petrolia and Veroia showed a significantly inferior performance (Figure 6). The correlation of their antioxidant activity (either FRAP or DPPH) was checked with their TPFC, TPHCl, THAD, TFLVN, and TFL contents. The best correlation was found for the aqueous preparations between the FRAP and TPFC data (Figure 7) with an equation *y* = 58.93*x* − 473.84 and *R^2^* = 0.92; where *y* = FRAP and *x* = TPFC. The lower correlation of DPPH scavenging activity with TP content of the studied extracts (MeOH: *R^2^* = 0.78 and EtOH: *R^2^* = 0.59) was attributed to the fact that five out of the eight extracts examined (with TP values for MeOH: 18–31 mg/g dry leaf and EtOH: 15–26 mg/g dry leaf) exhibited very high % inhibition values (MeoH: 89–92% and EtoH: 91–94%) that did not allow direct discrimination between samples [20]. According to Nenadis and Tsimidou [20], the relative concentration of the tested antioxidant is very critical and high antioxidant/[DPPH•] ratios resulting in % Inhibition values >80% do not permit observations for samples differentiation. 

### 3.5. Comparative Study of the Phenolic Bioactive Content of Olive Leaves from Two Sampling Periods

**Figure 8:**
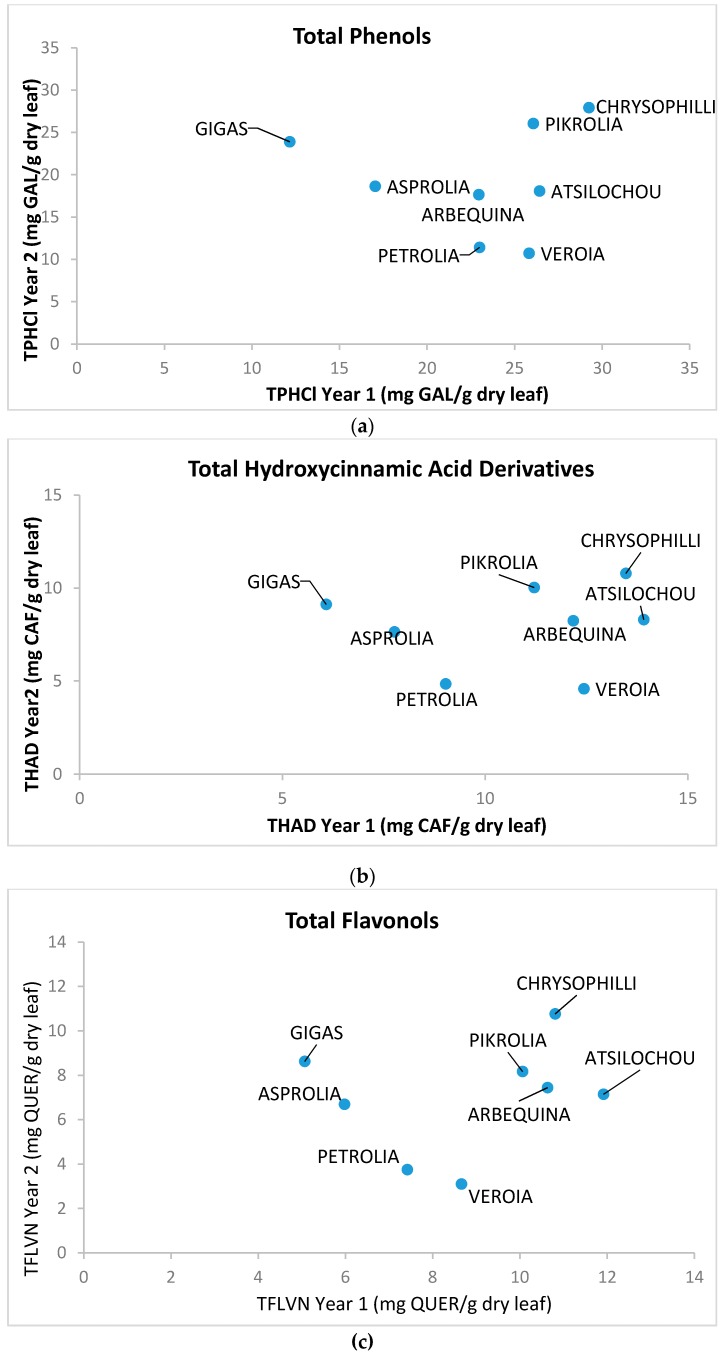
Comparative representation of (**a**) total phenol content determined via the alternative spectrophotometric assay (TPHCl), (**b**) total hydroxycinnamic acid derivatives (THCAD), and (**c**) total flavonols (TFLVN) data for the methanol extracts prepared from olive leaves from two sampling periods: year 1 (2012) and 2 (2013).

**Figure A1:**
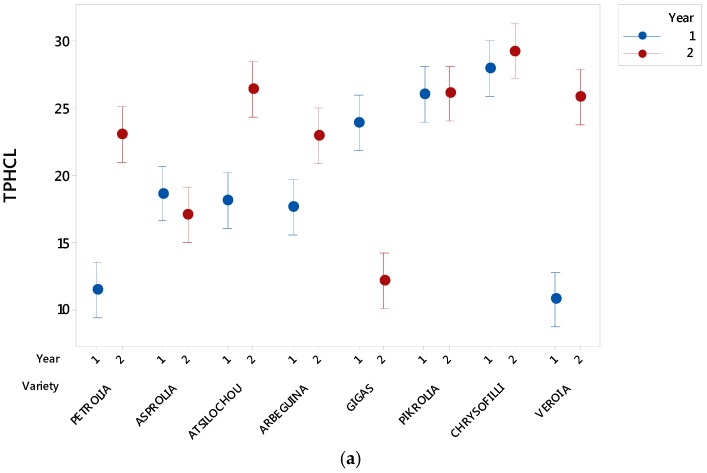

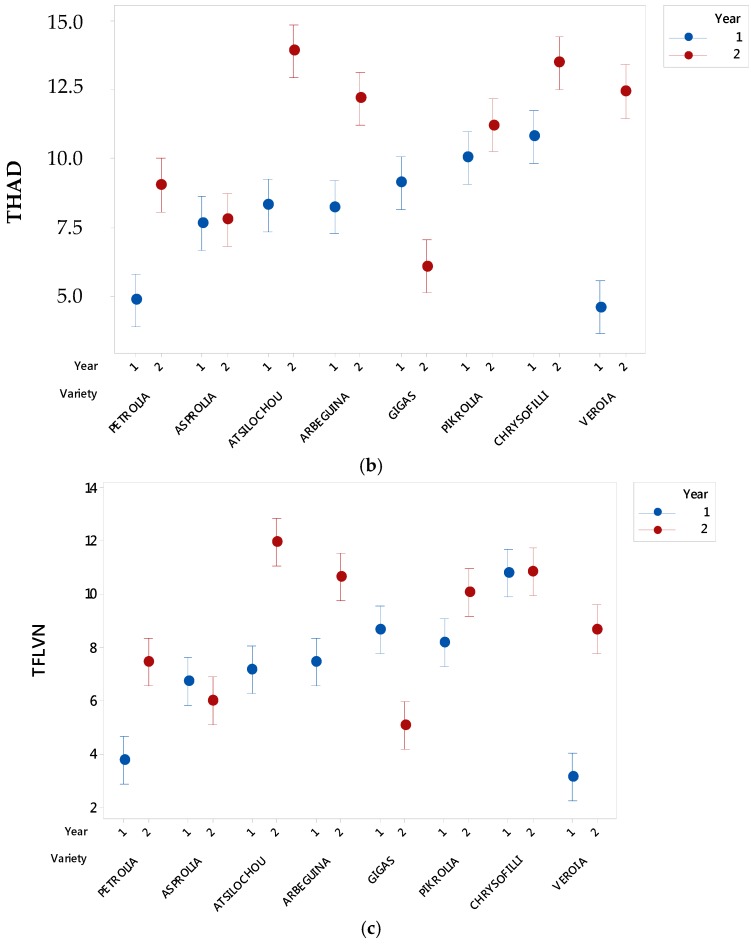
Effect of year and variety on total phenol content determined via the alternative spectrophotometric assay (TPHCl), total hydroxycinnamic acid derivatives (THCAD), and total flavonols (TFLVN) contents. Vertical bars denote the 95% confidence intervals of means calculated from the error mean square of the analysis of variance. Means whose 95% intervals do not overlap differ statistically significantly.

As presented (Figure 1, Figure 2, Figure 3, Figure 4, Figure 5 and Figure 6) and discussed in the previous sections, statistically significant differences are shown between cultivars that allow cultivars classification in high and low dynamic ones. The fact that cultivar affects the final quality characteristics of the material was expected and has been already discussed elsewhere [3,12]. In the present study, selective quality characteristics were re-examined in olive leaves collected from the same orchard for all studied cultivars in a second time-period. Data for the TPHCl, THAD, and TFLVN content for samples regarding the two examined periods are comparatively presented in Figure 8. Results depict that the range of bioactive class content was similar for the two sampling periods; however, the trend was not kept within varieties. Therefore, and as can be seen from Figure 8, Gigas cv., which was among the richer ones regarding TP, THAD, and TFLVN in the first sampling year, had lower bioactive content in the second sampling period. Moreover, Veroia cv. with poor TPHCl, THAD, and TFLVN contents in the first sampling period, presented high contents of the examined parameters in the second year of sampling. Similar were the observations for the rest of the studied cultivars (see Figure A1, where statistically significant differences are depicted). This finding was in line with published data revealing that even trees of the same cultivar grown under the same soil–climatic conditions can complete their developmental stage in a different way which may result in differences in total and individual phenol content and composition at specific collection times [3,12]. Moreover, the same cultivar may present a richer or poorer phenol content in a different sampling period [3,12]. Therefore, cultivars cannot be finally classified as being of high or low potency but they were all considered to have similar phenolic antioxidant content. This was seen as a challenging opportunity for the valorization of olive leaves coming from all cultivars, even those that are used as ornamentals and/or are not profitable for the olive oil or table olives sector. 

## 4. Conclusions

Data from one sampling period led to olive leaf cultivars classification as being of lower or higher bioactivity as regards their content in TP, THAD, TFLVN, TFL, and antioxidant activity (DPPH and FRAP). However, data from the second sampling period depicted that although the range of bioactive class content was similar for the two sampling periods, the trend was not kept within varieties. Therefore, all the studied cultivars were considered rich and equally potent for the studied bioactive parameters. This was seen as a challenging opportunity for the valorization of olive leaves irrespectively to cultivar origin. Moreover, although statistically significant different, the employed solvents presented of the same size potency to extract TP, THAD, and TFLVN. Methanol extracts presented significantly higher TFL values in relation to respective aqueous and ethanol ones. Last but not least, the HCl spectrophotometric assay was considered similarly effective with the widely employed FC for TP content determination. TP data derived from the two assays correlated well for the three solvents employed, encouraging the simple, rapid and inexpensive use of the HCl protocol in routine analysis of olive leaves polar extracts. 

## Figures and Tables

**Table 1:** Characteristics of the olive cultivars employed in the present study [14].

Cultivar	Use	Drupe Size	Location/Origin	Harvest Period	Particular Characteristics
Arbequina	Oil	Small	Spanish commercial cultivar, Globally known	Middle November–Middle December	Very productive; Resistant to cold; Moderately resistant to saline water, verticillium, & cycloconium; Suitable for super-high density planting linear cultivation systems
Asprolia	Ornamental	Small	Plant collections, Greece	Late November–Middle February	Average productive; Moderately resistant to cycloconium & cancer; Sensitive to cold
Atsilochou Ntopia	Oil	Small–medium	Central Peloponnese, Greece	November–December	Productive; Resistant to cold, winds & cancer; Moderately sensitive to olive fly & cycloconium; Suitable for high altitudes
Chrysophilli Epilogis	Ornamental	Small	East Africa, South Asia, Greece	November	Sensitive to cold. Sensitive to environments near sea; Resistant to dryness, winds, olive fly, cycloconium & cancer
Gigas Mastoeidis N.K.	Oil	Small–medium	East Peloponnese, Greece	Late November–Middle January	Productive when watered; Resistant to cold, cancer; Moderately resistant to cycloconium
Petrolia Serron	Oil Table Olives	Medium	Serres, Central & East Macedonia, Greece	Late November–Late December	Medium productive; Medium resistant to cold; Sensitive to olive fly & cycloconium
Pikrolia Corfu	Oil Ornamental	Medium	Corfu island, Greece	November–December	Low productivity; Resistant to poor soils, cold, cancer & cycloconium
Veroia Ntopia	Oil	Medium	Pieria mountains, Greece	November–December	Low productivity; Resistant to cold, snow & cycloconium

## Appendix

**Table A1:** Content of olive leaf (OLF) aqueous (H_2_O), methanol (MeOH), and ethanol (EtOH) extracts in Total Phenols (via the Folin–Ciocalteu assay: TPFC and via the Obied et al., assay: TPHCl), Total Hydroxycinnamic Acid Derivatives (THAD), Total Flavonols (TFLVN), Total Flavonoids (TFL), and antioxidant activity (caffeic acid equivalents of ferric reducing capacity (FRAP) for the aqueous and % DPPH radical scavenging inhibition for the alcoholic extracts).

Variety	TPFC		TPHCl		THAD		TFLVN		TFL		Antioxidant Activity	
Bioactive Parameter	mg caffeic acid/g dry leaf	SD	mg gallic acid/g dry leaf	SD	mg caffeic acid/g dry leaf	SD	mg quercetin/g dry leaf	SD	mg quercetin/g dry leaf	SD	FRAP: Caffeic acid equivalents or DPPH: % Inhibition	SD
**H_2_O OLF extracts**												
PETROLIA	12.52	1.31	10.71	0.50	3.51	0.47	1.87	0.63	0.87	0.50	272.58	19.38
ASPROLIA	19.39	0.40	14.78	1.59	5.32	0.97	3.17	1.11	2.33	0.11	672.31	68.85
ATSILOCHOU	21.69	1.10	20.92	1.56	10.12	2.73	5.87	1.11	3.97	0.18	1031.47	46.08
ARBEQUINA	12.87	0.47	12.95	2.23	5.06	1.03	3.34	1.25	1.85	0.15	183.14	87.15
GIGAS	25.71	1.88	19.60	1.55	7.15	0.31	4.77	0.29	2.15	0.33	888.14	33.23
PIKROLIA	27.40	0.96	26.07	1.72	11.92	1.53	8.53	2.64	3.95	0.55	1046.19	58.49
CHRYSOPHILLI	29.31	1.29	22.12	2.85	8.62	0.53	5.31	0.64	5.59	0.28	1335.08	59.47
VEROIA	11.67	0.34	10.17	1.16	4.28	0.08	1.73	0.24	1.82	0.37	241.50	82.11
**MeOH OLF extracts**												
PETROLIA	9.31	0.72	11.41	0.63	4.84	0.33	3.75	0.29	11.22	0.49	52.42	2.27
ASPROLIA	17.57	0.60	18.63	2.04	7.64	1.03	6.69	1.03	11.05	0.78	92.42	0.58
ATSILOCHOU	21.58	0.10	18.07	2.38	8.30	1.59	7.14	1.63	15.44	0.26	93.58	0.33
ARBEQUINA	12.80	0.22	17.64	0.82	8.24	0.33	7.44	0.35	13.20	0.52	65.47	0.59
GIGAS	22.36	0.22	23.89	1.91	9.11	1.00	8.62	1.07	16.31	0.68	91.60	1.05
PIKROLIA	21.54	0.23	26.04	4.47	10.02	1.15	8.17	0.86	13.66	1.18	90.97	0.39
CHRYSOPHILLI	30.57	0.35	27.93	2.67	10.79	1.69	10.76	1.58	22.54	0.06	92.92	0.33
VEROIA	7.15	0.11	10.72	1.12	4.58	0.44	3.10	0.25	4.93	0.38	32.23	2.72
**EtOH OLF extracts**												
PETROLIA	11.00	0.89	8.00	1.90	3.22	0.88	2.43	0.92	1.43	0.11	33.75	4.63
ASPROLIA	15.00	2.21	12.04	0.52	4.74	0.20	3.86	0.16	3.66	0.24	91.28	0.42
ATSILOCHOU	17.00	1.36	20.25	1.99	12.96	0.35	14.73	0.95	4.08	0.45	92.14	0.21
ARBEQUINA	11.00	1.30	10.18	0.66	4.66	0.34	3.84	0.35	3.36	0.13	56.43	9.95
GIGAS	27.00	1.05	18.45	0.34	7.04	0.22	6.20	0.27	9.26	0.07	90.59	1.07
PIKROLIA	20.00	1.30	23.36	2.72	8.95	0.33	8.36	0.95	8.20	0.23	88.93	1.05
CHRYSOPHILLI	26.00	1.34	23.82	0.35	9.51	0.12	8.87	0.14	8.49	0.25	91.38	1.00
VEROIA	9.00	1.18	7.42	1.03	3.01	0.47	1.94	0.46	2.54	0.30	33.13	5.41

SD: standard deviation.
